# Supplementary material for: Subglacial precipitates record Antarctic ice sheet response to late Pleistocene millennial climate cycles
Source: Nat Commun. 2022 Sep 15;13:5428. doi: 10.1038/s41467-022-33009-1 (PMC9477832; doi:10.1038/s41467-022-33009-1)
Supplement: Supplementary file 1 — Supplementary Information [file 41467_2022_33009_MOESM1_ESM.docx]

**Supplementary Information for “Subglacial Precipitates Record Antarctic Ice Sheet Response to Late Pleistocene Millennial Climate Cycles”** by G. Piccione, T. Blackburn, S. Tulaczyk, E.T. Rasbury, M.P. Hain, D.E. Ibarra, K. Methner, C. Tinglof, B. Cheney, P. Northrup^2^, K. Licht

**Supplementary methods**

^234^U-^230^Th dates were produced at the University of California Santa Cruz (UCSC) Keck Isotope Laboratory. Samples were spiked with a mixed ^229^Th-^236^U tracer that was calibrated against a gravimetric U-Th solution for isotope dilution analyses. They were then digested in 3mL 7N HNO_3_ (calcite) or concentrated 4mL HF + HNO3 (opal) via benchtop dissolution and dried down. U and Th separates were purified using ion chromatography with 1mL columns of 200-400 mesh, AG1-X8 anion resin. Samples were loaded onto the column in 1mL of 7N HNO_3_ and major elements were washed off with an additional 2ml HNO_3._ ­Loading and washing eluant was collected and saved for Sr analyses. Thorium was eluted in 2mL of 6N HCl. Uranium was then eluted in 2mL of ultra-pure water. This column procedure was then repeated to achieve U and Th purity levels necessary for analyses. Total procedural blanks were <10pg for U and <25pg for Th, which are minor relative to sample concentrations. Both U and Th isotopic measurements were conducted using the IsotopX X62 Thermal Ionization Mass Spectrometer (TIMS) housed at UCSC. U and Th samples are loaded onto 99.99% purity Re ribbon. Uranium is loaded in a Si-gel activator and measured as UO_2_. Uranium compositions were corrected for oxide isobaric interferences following ref.^1^. Uranium measurements were performed as a two sequence “Fara-Daly” routine: in the first sequence, ^234^U (mass 266) is collected on the Daly, while ^235^U (mass 267) and ^238^U (mass 270) is collected on the high Faraday cups equipped with 1e^12^ Ω resistors. The second sequence placed ^235^U (mass 267) on the Daly and ^236^U (mass 268) and ^238^U (mass 270) on the high Faraday cups. The 266(Daly)/270(Faraday) composition was corrected using the Fara-Daly gain: (267Faraday/270Faraday) / (267Daly/270Faraday). Uranium compositions were corrected for oxide isobaric interferences following ref.^1^. Mass dependent fractionation correction was applied using a linear correction with correction factor determined from long-term measurement of standards. Uranium dead times for the Daly were calibrated using NBS U-500. Accuracy of the uranium method is evaluated using Uranium standard NBS4321 (Supplementary Fig. 8). Thorium isotope measurements were also done on the TIMS at UCSC. Thorium is loaded in a graphite emitter and measured as a metal. Each mass of Th is measured using a peak hopping routine on the Daly. Thorium fractionation and deadtime were estimated by running NBS U-500 as a metal. Accuracy of ^234^U-^230^Th dates were tested using MIS 5e coral and compared to dates in ref ^2^, as well as a previously dated carbonate precipitate^3^. U-Th ages are calculated using codes designed at UCSC. All ages are corrected for initial [^230^Th/ ^232^Th] assuming a composition of 4.4±2.2e-6. As the exact [^230^Th/ ^232^Th]_i_  is unknown, we assume this ratio from the expected composition of the silicate upper crust in secular equilibrium, allowing for a departure from this composition of 50%, and propagating this uncertainty through to the final age. Decay constants for all data and models were from ref. ^4^. All uncertainties are reported at 2σ, unless otherwise specified.

**Supplementary Note 1**

In the main text, we have briefly discussed the potential source areas of the two precipitate samples: MA113 and PRR50489. Here, we provide further relevant details.

Sample MA113 is found at Mount Achernar Moraine (henceforth MAM; 84.2°S, 161°E), a nearly motionless body of blue ice on the side of Law Glacier, with average surface velocity of about 25 m a^-1^ ^7^. The moraine is located ca. 20 km downstream of the polar plateau, and its debris is derived locally, with Beacon and Ferrar Group rocks dominating the sample collection area. The subglacial origin of the geologic material is supported by an abundance of striated and faceted clasts^8^. Subglacial debris is transported to the surface of MAM with upward-flowing ice from the depths of Law Glacier, and is accumulated on the surface as the surrounding ice sublimates (e.g., figure 9 in ref. ^8^). Graly et al. (2018) inferred that the most plausible mechanism of subglacial entrainment of the debris is regelation in an open hydrological system and assessed that suitable conditions for this process exist 30-50 km upstream from the moraine^9^. The length of time for the emergence of basal debris to MAM is estimated to be at least 35 ka^8^, which is close to the youngest radiometric U-series age obtained for sample MA113 (25.44 ± 0.59 ka). The similarity between the two timescales supports the possibility that the sample formed in a location much more proximal to MAM than 30-50 km, perhaps in one of the overdeepenings or steps in bedrock topography in Law Glacier valley that can be found within several kilometers of the moraine (e.g., Figure 7 in ref. ^7^). Such a topographic depression represents suitable settings for precipitate formation because the bed geometry would allow subglacial waters to become isolated and overconcentrated to the point of opal precipitation during regional basal freezing periods and millennial cold phases. Calcite precipitation may occur as the regional basal water system reconnects to topographic depression during the regional expansion of basal melting, which is associated with warm AIM phases in our model (Fig. 6). Whereas we cannot pinpoint the exact location from which the sample MA113 originated, we conjecture that it was formed in a subglacial topographic depression located several kilometers to a few dozens of kilometers upstream of MAM.

Sample PRR50489 comes from Elephant Moraine (henceforth EM; 76.3°S, 157.3°E), a supraglacial moraine in a blue ice area of Transantarctic Mountains, where ice sublimation persisting for estimated ~100 ka released debris from basal ice of the East Antarctic ice sheet^10,11^. The current sublimation rate in the area is ca. 0.04 m/year ^12^. It is not yet proven what triggered the formation of EM, but it could have been related to a capture of the upper part of Mawson Glacier drainage by the southern tributaries of the David Glacier, which experienced vertical incision of hundreds of meters in the last 234 kyrs^13^. This switch from eastward ice flow towards the modern Mawson Glacier to northward flow towards David Glacier could have aided the emergence of basal ice layers at the EM. In the discussions below, we will assume that at the time of PRR50489 formation, which predates the formation of the EM itself, ice upstream of the moraine was flowing towards Mawson Glacier. However, none of our fundamental inferences would change significantly if, instead, we assumed that the modern ice flow pattern (i.e., ice flow from EM north towards David Glacier) prevailed then.

Rocks found on the surface of EM include material from the Beacon and Ferrar Supergroups and some Tertiary fragments with Neogene marine microfossils, interpreted to have come from the subglacial Wilkes Basin located to the west of the mountain range^14^. We use the simple method employed in ref. ^7^ to estimate the time needed for our subglacially formed sample to emerge from the bed through 1.5-2.0 km of ice, which are the typical ice thicknesses upstream of EM in the Bedmap2 dataset^15,16^. To approximate the emergence timescale, we divide ice thickness by the sublimation rate and get a range of ca. 40-50 ka. To glean information about the precipitate source area from this emergence timescale, we must also account for the time that the sample may have spent laying on the surface of EM. Cosmogenic surface exposure ages of EM boulders were measured to be up to 60 ka ^17^, and meteorites found nearby have exposure ages up to 100s of thousands of years^18^. Given the significant uncertainties associated with the emergence timescale and the surface-residence timescale for this sample that was still precipitating in subglacial water <145 kyr ago, we conservatively assume a wide temporal range, 10-100 kyr, for the period it spent both traveling horizontally in basal ice and sitting on top of the moraine. Whereas the ice surface velocity over EM itself is very slow (<0.1 m/yr)^10^, the proximal upstream part of the southern drainage area of David Glacier moves at ca. 5 m/yr ^19^. Using the modern flow configuration^20,21^, the longest ice flowline reaching EM from the regional ice divide separating drainage basins of David and Mulock Glaciers is ca. 150 km. In theory, our sample could have traveled from this area if its average velocity over our assumed upper bound for horizontal travel (100 kyrs), was 1.5 m/yr. However, this is likely to be too large an average basal transport velocity to assume over such an extended period, which includes cold glacial conditions with accumulation rates, and hence also balance ice velocities, that were 2-3 times lower than Holocene rates^22^. In addition, a debris fragment embedded in basal ice travels at velocities lower than the surface ice velocity. For instance, in their analysis of basal debris transport to MAM, Kassab et al. (2019) assumed an average transport velocity of 0.5 m/yr even though ice surface velocities within Law Glacier are about 25 m/yr ^7^. In the slower flowing region (surface velocity of 5 m/yr) upstream of EM this would be equivalent to basal transport velocity of 0.1 m/yr. At such speed, our sample would have traveled only 1-10 km in the assumed period of horizontal transport ranging from 10 to 100 kyrs. We conjecture that PRR50489 formed in a basal overdeepening within a relatively small range away from EM. Although the horizontal resolution of Bedmap2 bed topography is worse here than in the vicinity of MAM, it contains an indication of a broad overdeepening that starts a few kilometers upstream of EM

**Supplementary Note 2**

It is conceivable that the cyclicity in opal and calcite result from melting of compositionally distinct basal ice proximal to the precipitate formation location, rather than from geographically distinct water sources. For this explanation to be viable, would require that the opal forming basal ice be free of dissolved carbon, to prevent calcite formation upon freezing requisite for opal saturation. Basal ice, however, is noted to be highly enriched in carbon relative to meteoric ice^23–25^. A second requirement is that calcite forming basal water need have δ^18^O compositions more ^18^O-depleted than any known ice in Antarctica. The δ^18^O compositions of sample MA113 (-61.2‰) is more ^18^Ο-depleted than any known ice in Antarctica, likely meaning that this endmember water experienced some degree of freezing in transit to the ice margin. A plausible scenario begins with ice melting beneath the wet based portion of the EAIS, 20km up glacier from MA, such as the -55 to -59 ‰ δ^18^O compositions observed within the South Pole ice core^26^. Those waters, as they migrate from the thick, wet-based portion of the EAIS towards the margins, will undergo freezing, further depleting the δ^18^O compositions of the water and forming basal ice that is *enriched* relative to the initial melt^27^. Collectively, these requirements suggest that both opal and calcite forming endmembers brought to the precipitate formation location in the aqueous phase, rather than as basal ice.

A second alternative precipitate formation mechanism was presented by Faure and others^28^ to explain EM precipitates, and proposes that opal-calcite samples formed in subglacial hotsprings when solute-rich hydrothermal groundwater interacted with melted basal ice. This explanation was made before the discovery of widespread subglacial water and brines at the base of the EAIS, when authors would have had to assume that hydrothermal groundwater is required for highly saline fluid to be present at the base of the ice sheet. We greatly expand on the geochemical characterization presented in ref. ^28^, add chronologic data to the EM precipitates, and find a similar subglacial precipitate from Law Glacier that forms via the same mechanism. These new data require two geographically distinct waters to mix on timescales matching SH millennial-scale climate cycles. In the following paragraph, we outline geochemical evidence that opposes a hydrothermal origin for these opal-calcite precipitates.

The two precipitates presented in this manuscript have isotopic compositions of carbon, oxygen, and uranium that would be highly improbable in the hydrothermal formation scenario presented in ref. ^28^. The δ^13^C of the calcite endmember water for both samples clusters tightly at a highly negative value (ca. -23‰ for PRR50489 and ca. -18‰ for MA113). As described in the main text, this composition requires that microbes in the aqueous system selectively oxidized the most readily available carbon source (likely fossil terrestrial organic matter). Such uniform δ^13^C values have been found in sediment-rich basal ice beneath the AIS^23,25^, and have been hypothesized in subglacial aqueous environments^29,30^. If a hydrothermal water supplied carbon to the aqueous system, the δ^13^C of that carbon would be similar to TAM soils (0.2 – 8.5‰ ^31^) and therefore offset by 23 – 31‰ from the ~ -23‰ values measured in subglacial precipitates. Likewise, the δ^18^O value of calcite-endmember water in both samples also appears too low to reflect a hydrothermal source. For sample PRR50489, the calcite-endmember δ^18^O value (-55.3‰) is close to the most ^18^O-depleted value observed in ice up glacier from EM (-56‰; Dome-C^32^), meaning that, if we conservatively assume that hydrothermal waters have a δ^18^O between -10‰ and 10‰, >98% of the water in the subglacial system would have to come from fresh glacial meltwater. However, Antarctic meteoric ice is dominated by marine Sr and U isotopic compositions^33^. Thus, an influx of melted glacial ice would violate Sr and U isotopic values of the two samples, which instead require long-term water rock interaction^34^. What is more, the δ^18^Ο value of the calcite endmember water from MA113 is -61.2‰, which is more depleted than any ice upstream of Law Glacier (South Pole Ice Core^26^). It is likely that this extremely low δ^18^Ο value results from some portion of the calcite-endmember water experiencing freezing prior to mixing to form the calcite layer. For this δ^18^Ο value to result from Faure’s hydrothermal mechanism would require basal ice that is even more depleted than -61.2‰, which is highly unlikely given that it has never been observed in an Antarctic ice core. Finally, Enrichment in ^234^U is consistent with a surface water and would be rare for a hydrothermal water. The extreme (500%) ^234^U enrichment observed in our precipitates occurs from prolonged (>10 ka) water-rock interaction at the ice sheet-substrate interface. While hydrothermal water can achieve this composition following prolonged leaching of bedrock, mixing with melted glacial ice would dilute this signal with a ^234^U/^238^U signal of 1.14 ^33^. Subglacial waters with elevated ^234^U/^238^U are not rare in Antarctica. Similar compositions are observed in Blood Falls brine^35^, Don Juan Pond^36^, and Antarctic Precipitates found at Lewis Cliff Glacier^37^, the Pensacola Mtns.^38^, Law Dome^39^ and Boggs Valley^3^. Perhaps most importantly, precipitates from the Laurentide ice sheet^40^ and Yosemite Valley^41^, areas that are not volcanically active, also record ^234^U enrichment. This prevalence of ^234^U enrichment in subglacial waters across a wide range of space and time is an indication that long-term residence of water beneath ice sheets is a common occurrence.

In summary, our geochemical observations from precipitates MA113 and PRR50489 provide evidence that opal and calcite layers formed from mixing of two different fluids, glacial meltwater and brine. These fluids have compositions that match waters readily found beneath ice sheets, even in volcanically inactive areas. Uranium series dates from both samples demonstrate that mixing of these two fluids occurs contemporaneously with millennial-scale SH climate cycles. A formation mechanism therefore calls on a glaciologic process for driving subglacial hydrologic changes throughout the Ross Embayment.

**Supplementary Note 3**

We use a reduced complexity model of ice sheet thermodynamics to demonstrate that there is a glaciologically plausible mechanism for explaining the two key observations derived from the precipitate samples MA113 and PRR50489: (i) cyclic opal-calcite precipitation from subglacial waters switching between oxygen- and carbon-poor brines and carbon- and oxygen-rich meltwaters, and (ii) the correlation between opal precipitation and millennial cold phases, and between calcite layers and millennial warm phases. Combined, these observations indicate that the subglacial water bodies in which these two samples formed, although separated by nearly 1000 km distance, experienced hydrologic isolation and cryoconcentration during millennial cold phases and became open to basal meltwater inputs during warm phases. Glaciologically, the most parsimonious explanation is that the cold millennial phases corresponded to basal freezing conditions, while warm phases to basal melting conditions in the two areas of sample formation.

Our modeling approach is inspired by the simplicity of the binge-purge model of Heinrich events^42^. In fact, initially we considered the binge-purge model to be a potential explanation for the millennial-scale opal-calcite couplets found in our samples due to the shared millennial-scale periodicity of both. However, the fundamental problem with this interpretation is that the binge-purge model predicts millennial-scale switches between basal freezing and melting for the Hudson Bay ice stream assuming surface accumulation rates that are about one order of magnitude higher than the accumulation rates prevailing in our Antarctic study areas^42–44^. Hence, application of the binge-purge scaling (right-hand side of equation 12 in ref. ^42^) with such small accumulation rates would yield unsatisfactorily long periodicity. Moreover, it would be a remarkable coincidence if a climatically unforced binge-purge oscillator, as proposed in ref. ^42^, would result in the observed correspondence between the periods of opal and calcite precipitation, and cold and warm phases of AIM cycles at two locations very distant from each other (Fig. 1). Reconstructions of recent internally driven ice stream cyclicity in West Antarctica yield periodicities of hundreds of years, which are in turn much shorter than the AIM-scale ice dynamic variability needed to explain our observations^45^. Given these considerations, we formulate the RCMIST to illustrate a glaciologically plausible link between millennial-scale climate forcing and subglacial precipitation of opal and calcite driven by changes in basal thermal regime. It is crucial to keep in mind that this model is used here solely for illustrative purposes. There are simply too many relevant observational uncertainties (e.g., locations of sample precipitation, geothermal flux at these locations, ice thickness and ice flow history during sample precipitation, etc.) to constrain a more complex model aimed at reconstructing in detail ‘what really happened’ during sample precipitation. Instead, our goal is to illustrate a plausible mechanism with a simple model.

Switches between basal melting and freezing conditions are controlled by the basal thermal energy balance, *E*. The three most fundamental controls on E are two sources of heat (*G* = geothermal heat flow and *S* = shear heating accompanying ice motion), and one sink of heat (*Q* = conductive heat loss) (e.g., ref. ^46^):

*E=G+S-Q* Eq. 1

There is no physical reason for geothermal heat flow to vary on the timescale of AIMs. Hence, we treat it as a time invariable parameter with a value of 0.05 W/m^2 47^. Changes in surface temperature and accumulation rate accompanying AIMs can impact the basal thermal energy balance through the conductive heat loss term, *Q*, but the ice sheet does mute this effect through^42^: (i) dampening the amplitude of temperature variations with depth, and (ii) introducing a time lag between surface climate forcing and basal thermal response. In ref. ^42^ authors pointed out that a under the purely conductive vertical heat transport, a periodic surface temperature forcing will decay exponentially with e-folding depth scale of 314 m. Given that the potential sample source areas have ice thicknesses of about 1500m^15,16,48^, an AIM-scale surface temperature fluctuations of 1-3°C^49,50^ would amount to 0.008-0.025°C change in ice temperature near the bed. Thicker ice would cause even more attenuation of the temperature signal and much thinner ice thickness (e.g., 1000m) would result in our model always predicting basal freezing, rather than switches between melting and freezing conditions at the ice base. Using equation 1 from ref. ^51^ we calculated that, even assuming an instantaneous thermal equilibration throughout ice thickness, the maximum difference between the conductive heat loss during cold and warm AIM phases would be about 0.001 W/m^2^ (assuming ice thickness of 1500m, temperature change of 3°C, and accumulation rate of 0.03 m/yr during the warm phase and 0.02 m/yr during the cold phase^52^.) This is more than an order of magnitude less than the assumed geothermal flux. We will show later that it is also one to two orders of magnitude less than the contribution from the shear heating term, *S*.

The second fundamental problem with relying on vertical advection and diffusion of surface climate signals to the bed is the significant time lag with which such a transfer happens (e.g., ref. ^42^). The timescale for purely conductive heat transfer can be estimated from the ratio of the square of ice thickness (*H*) to the thermal diffusivity of ice, which for *H* of 1500 m and diffusivity of 44 m^2^/yr ^42^ yields ca. 50,000 years. The equivalent timescale for the purely advective vertical heat transfer can be approximated as the ratio of the ice thickness to the surface accumulation, which for reasonable assumptions of 1500m and 0.03 m/yr ^52^, respectively, also yields 50,000 years. The low accumulation rates assumed for the regions of interest are justified by both ice sheet modeling of ice sheet sensitivity to AIM forcing^48^, and the fact that the two samples were found in locations where under modern conditions the surface mass balance is negative^7,53^. These low surface accumulation rates are also compatible with recent direct observations in the study regions^44^ and with reconstructions of accumulation rates from the Taylor Dome ice core^43^. Although we have used one specific thickness, 1500 m, to calculate these hypothetical timescales, our results would not substantially change if we would consider a wider range of plausible thicknesses, e.g., 1000-2000 m, for which these timescales would also be in tens of thousands of years. Given that these diffusive and advective time scales are about an order of magnitude longer than the millennial-scale AIM climate fluctuations, we do not favor variations in the conductive heat loss term (*Q* in equation 1) as an explanation for the cyclicity in subglacial hydrological conditions inferred from our samples.

By the process of elimination, we arrive at the shear heating term, S, in equation 1 as the most promising mechanism for triggering switches between basal melting and freezing conditions on millennial timescales. Given the slow ice motion at, and upstream of, sample collection locations^16^, we approximate the shear heating term, *S*, as a product of the driving shear stress and deformational ice velocity, *U*, averaged over ice thickness:

$S=\tau U=\frac{2A}{n+2}\tau^{n+1}H=\frac{2A}{n+2}\left( \rho g\alpha\right)^{n+1}H^{n+2}$ Eq. 2

where $\tau=$gravitational driving stress ($\rho gH\alpha$), $\rho=$ ice density, $g=$ gravitational acceleration, $H=$ ice thickness, $\alpha=$ ice surface slope, *n* = stress exponent in the ice flow law (assumed to be 3, ref. ^54^, table 3.3), *A* = ice viscosity parameter (the value for ice at 0°C in the table 3.4 in ref. ^54^). We follow the simplifying assumption that all shear heating can be attributed to ice motion at/near the basal interface^55^. Equation 2 is based on shallow ice approximation assumption for an ice sheet moving through internal ice deformation. These assumptions are justified based on the relatively modest ice surface velocities dominating the two sample collection areas, MAM and EM, and the regions that feed ice into them from upstream^16,20^.

Equation 2 contains two glaciological variables that vary with climate forcing, the ice surface slope and ice thickness. Since the latter is raised to a higher power ($H^{5}$) than the former ($\alpha^{4}$), perhaps ice thickness is the preferred pathway through which millennial scale climate changes have influenced shear heating in the two sample formation areas? However, to match the observed precipitation patterns, ice thickness would have to increase during warm AIM phases and decrease during millennial cold phases. The only published model of Antarctic ice sheet sensitivity to AIM climate forcing^48^ shows the ice sheet thickness decreasing with increasing temperature in both regions from which our samples have been collected. This suggests that the tendency for the ice sheet to thicken as accumulation rate increase under warming climates is overcome by an increase in the dynamic ice thinning associated with the grounding line retreat and ice flow acceleration during warm AIM phases^48^. This is certainly consistent with the fact that ice thicknesses decreased by close to 1000 m in Transantarctic Mountains^56,57^ in response to climate warming after the last glacial maximum even though accumulation rates roughly doubled^22^. Similarly, Neuhaus et al. (2021) proposed that the grounding line in the Ross Embayment retreated when accumulation rates increased and advanced when they fell during Holocene millennial-scale climate variations^58^.

The dynamic effect driving ice sheet evolution in response to ocean thermal forcing on grounding lines is incorporated into our simplified model of shear heating (Equation 2) through the ice surface slope, which steepens when the ice in the Ross Embayment thins during grounding line retreats (AIM warm phases) and becomes shallower when ice sheet thickness in the Ross Embayment increases during grounding line advances (millennial cold phases). In our calculations of time-dependent shear heating we parametrize the evolution of regional ice surface slope along an ice drainage pathway connecting a sample origination region to the Ross Embayment:

$\alpha(t)=\left[ \Delta b+H(t)-H_{RE}(t) \right]/L_{o}$ Eq. 3

where $\Delta b=$ bedrock elevation difference between the region from which a sample originated and the part of the Ross Embayment into which ice from this region is draining (at the foothills of Transantarctic Mountains), $H\left( t \right)=$ ice thickness in the region of sample origin, $H_{RE}\left( t \right)=$ ice thickness at the foothills of Transantarctic Mountains (e.g., locations of the mouths of Law Glacier for the MAM sample and Mawson Glacier for the EM sample), $L_{o}=$ a length scale representing the distance between the sample origination region and the ice discharge area in the Ross Embayment. Consistent with the simplicity of our model we assume $L_{o}$ to be ~100 km, which is approximately equal to the distance between MAM and EM locations and the mouths of Law and Mawson Glaciers. The bedrock elevation difference is estimated from existing bed elevation datasets^15,16^ and our preferred locations of sample origin (see the discussion above). We have run multiple sensitivity tests assuming different values of $\Delta b$ and $L_{o}$and the results presented below and in the main manuscript are not fundamentally dependent on the preferred values stated here being exact. The model produces the desired switches between basal melting and freezing for wide ranges of $\Delta b$ and $L_{o}.$

Whereas the ice thickness evolution in the region of sample origin, $H\left( t \right),$is a variable calculated by the model from ice mass balance calculation (see Eq. 5 below), the ice thickness at the foothills of Transantarctic Mountains $H_{RE}\left( t \right)$is used as the forcing function that is driving the temporal response of the simulated system to climate forcing. The underlying idea is that the climate forcing, which is represented here by isotopic records from ice cores, is driving changes in the position of the grounding lines in the Ross Embayment and, hence also the changes in $H_{RE}\left( t \right).$Ice thickness forcing at the foothills of Transantarctic Mountains, $H_{RE}\left( t \right)$, is parametrized as a linear function of an ice core isotopic record of paleoclimate, $i\left( t \right)$, covering the time periods of sample precipitation:

$H_{RE}\left( t \right)=H_{o}+C_{i}\left[ i\left( t \right)-i_{o} \right]$ Eq. 4

where $H_{o}=$ is the initial thickness (taken from ice sheet model output of ref. ^48^), $C_{i}=$ proportionality constant with units of meters per ‰, and $i_{o}=$ the initial isotopic value in ‰. For simulations of basal thermal conditions pertaining to the sample MA113 we use the δ^18^O record from the WAIS Divide ice core^59^ and for the older sample PRR50489 we use the δD record from the EDC ice core^60^. In our model the ice thickness in the sample formation area, $H(t)$, evolves through time following this simple mass-balance ODE:

$\frac{\partial H(t)}{\partial t}=aL_{a}-UH(t)$ Eq. 5

where $t=$ time, $a=$ accumulation rate (0.02-0.03 m/year taken from the ice sheet model output of ref. ^48^), and $L_{a}=$ accumulation length scale (i.e., taken to be 500 km as the approximate length of the accumulation zone upstream of the site where $H(t)$ is evaluated. The first term on the right-hand side represents an aggregated influx of ice from upstream while the second term is an aggregated outflux of ice in downstream direction. It is important to keep in mind that in this flowline formulation of mass balance, the left-hand side of this equation is implicitly multiplied by unit width, which resolves the apparent mismatch in units between the two sides of Eq. 5. The accumulation length scale should not be taken literally (e.g., as the distance from the sample origination site to the ice divide), given the fact that there is considerable flow conversion occurring as ice flow funnels from widespread upper drainage areas towards relatively narrow valleys of outlet glaciers crossing the Transantarctic Mountains. Rather, $L_{a}$ is used in our model as a flowline representation of the upstream accumulation area. We examined the sensitivity of our results to different choices of $L_{a}$ ranging from dozens of kilometers to over 1000 km and got the desired oscillations in basal thermal conditions when $L_{a}$ ranges between ca. 300 and 800 km. For too short $L_{a}$ there was only basal freezing and for too long $L_{a}$ there was only basal melting. The accumulation rate is kept constant since our model sensitivity experiments indicated that varying it with time did not materially impact the model output.

One concern that can be raised about the applicability of the system of equations 4 and 5 is that they do not account for any lags between the climate forcing and the glaciological response at the sample origination sites. By neglecting these lags, we assume that they are smaller than the combined dating uncertainties of the ice core timescale and the U-series-dated sample precipitation chronology. In the case of MA113, the uncertainties in the sample age model, >1.5 kyr, are clearly higher than the uncertainties in the WAIS Divide ice core record, ~0.5 kyr ^50^. For the older sample, PRR50489, we use the EDC ice core record, which has considerably higher uncertainties, 2-4 kyrs, over the period of sample precipitation^61^. In general, a lag in the glaciological response to the climate forcing would have to be >2 kyrs, to justify its inclusion in our reduced-complexity model. Below we argue that plausible response timescales are shorter than this. Propagation of glaciological perturbations takes place through kinematic-wave and diffusive processes^62^, with the former dominating in low surface-slope areas moving through basal sliding (e.g., ice streams) and the latter in high surface-slope regions where ice motion is accommodated predominantly through internal deformation (section 11.3.3 in ref. ^63^). As a conservative example, we will consider here the case of the MAM study area, which is located further towards the ice sheet interior than the EM region and, hence, should have longer glaciologic response times to ocean thermal forcing. Our sample precipitated in this region, MA113, formed entirely during the Marine Isotope Stage 3 when climatic conditions were colder than during the Holocene but not as cold as during the last glacial maximum^64^. There are no firm constraints on the position of the grounding line in the Ross Embayment during MIS 3, so for simplicity we will assume that this grounding line was, on average, at the approximate position of the front of the modern Ross Ice Shelf, with fluctuations around this position during warm and cold millennial phases. This would put the point of discharge of Law Glacier into the Ross Embayment about 600 km away from the assumed average MIS 3 grounding line position. Fast flowing ice streams crossed the grounded ice in the Ross Embayment^65^ and we will assume that they moved with speed of ca. 0.3 km/yr, which would translate into kinematic wave speed of ca. 1 km/yr ^62^. In this scenario, glaciological effects of grounding line migration would take about 0.6 kyrs to arrive at the mouth of Law Glacier. The lower half of this glacier moves at present with high enough velocities, ca. 0.1-0.4 km/yr to infer that it is likely sliding. Hence, we will also use here the kinematic wave speed to estimate how long it would take for a glaciological perturbation at the mouth of the glacier to propagate half-way its length, i.e., ~50 km. With assumed average speed of ca. 0.2 km/yr, the kinematic wave speed is ca. 0.7 km/yr and the 50-km travel time is ca. 0.07 kyrs. The last ca. 50 km of the glacier towards the MAM experiences slow ice motion, 0.02-0.03 km/yr, suggesting dominance of internal ice deformation and diffusive processes for propagation of glaciological perturbations. Assuming average surface slope of 0.006, ice thickness of 1 km, and average ice velocity 0.02 km/yr ^16,20^, we estimate the horizontal diffusivity to be 10 km^2^/yr, and a diffusional timescale of signal propagation of 0.25 kyrs over 50 km. Altogether, we estimate that it would take a grounding-line perturbation about 1 kyr to propagate from the grounding line to the MAM region. We justify the fact that our equations 4 and 5 do not make a provision for this lag by the fact that 1 kyr is significantly shorter than the combined uncertainties in the chronology of our samples and of the isotopic ice core records used to force temporal variations in our model.

To produce the output shown in Figures 2e and 3e of the main manuscript text, we solved numerically the system of equations 1 through 5 using a forward-difference solver with time step of 10 years and a set of the necessary initial conditions. Each simulation was started 10,000 years before the beginning of the two precipitate records to provide model spin-up time for relaxation of the calculation from any artifacts associated with our choice of initial conditions. The basal thermal energy budget is expressed in terms of equivalent basal melting/freezing rate in units of mm/year using the volumetric latent heat of ice 3×10^8^ Joules/m^3^. The key tunable model parameter is $C_{i}$, the constant determining the sensitivity of ice thickness changes, $H_{RE}\left( t \right)$, to variations in isotopic records of paleoclimate (Equation 4). We used visual inspection of outputs from dozens of sensitivity runs to evaluate which values of $C_{i}$ yield satisfactory agreement with the geochemical record obtained on the two samples. The sensitivity of our results to $C_{i}$ is illustrated in Supplementary Figure 9a. For the set of control parameters used to generate Supplementary Figure 9a, the basal heat budget experiences switches between melting and freezing in the right time periods to explain changes between opal and calcite precipitation if the $C_{i}$ parameter is between 10 and 23 m per ‰. A $C_{i}$ value that is too low does not yield the expected melting-freezing switches in the last 15,000 years of the record (e.g., $C_{i}=5 m per ‰$ in Supplementary Figure 9a). A $C_{i}$ value that is too high results in output that predicts freezing period between 180 and 200 kyrs BP that are too long (e.g., $C_{i}=25$ m per ‰ in Supplementary Figure 9a). The satisfactory results, $C_{i}=$ 10 to 23 m per ‰, are consistent with ice thickness changes in the Ross Embayment, $H_{RE}\left( t \right)$, of a few hundreds of meters on the millennial scale of AIM climate cycles. This magnitude of ice thickness variations corresponds to the cases of high sensitivity of the Antarctic ice sheet to ocean thermal forcing in numerical experiments of ref. ^48^. Supplementary figure 10 shows equivalent results for the sample MA113, with $C_{i}=$ 10, 50, and 250 in units of m per ‰ of δ^18^O (since we are using the WAIS Divide ice core record for this sample). Generally, satisfactory results are obtained for MA113 when $C_{i}$ is between ca. 50 and 200 m per ‰. A $C_{i}$ value that is too low produces switches to basal freezing in the second half of the record that are too infrequent. While a $C_{i}$ value that is too high misses some switches to basal melting (e.g., Supplementary Figure 10). Ice thickness changes that produce satisfactory results are in the range of a few hundred meters variation in $H_{RE}\left( t \right)$. This result is also consistent with the ice sheet model runs of ref. ^48^ that assume high sensitivity to the ocean thermal forcing.

**Supplementary Note 4**

Here we present preliminary thin section images and elemental maps of PRR50489 and MA113 to characterize opal and calcite textures. The hypothesized hydrologic freeze-flush mechanism for opal-calcite formation may impart certain textural characteristics to the precipitates, including delivery of detritus to the system during flushing events (i.e., in calcite layers), changes in calcite crystal growth patterns, and evidence for detrital settling at the base of calcite layers. However, certain unknown physical parameters may preclude these textural characteristics from forming. Namely, the velocity of waters flushing during periods of subglacial connectivity is unknown and flushing events need not be catastrophic floods like those observed in active, marginal, subglacial hydrologic systems. Secondly, the timing of calcite formation, amount of sediment delivery, and frequency of flushing events during a given millennial-scale warm period are unknown and would affect the distribution of detritus within calcite layers. The amount of physically weathered detritus in the subglacial environments beneath the EAIS is likely lower in environments in the EAIS interior than in more marginal systems, where mechanical weathering is enhanced due to higher velocity ice flow^56^. Finally, we do not know the depth nor horizontal extent of the subglacial basins, which pertains to sediment content in our samples because detritus delivery during hydrologic flushing events could be concentrated near the mouth of the basin but would not necessarily be basin wide. Therefore, while textural evidence for water motion within MA113 and PRR50489 could lend more confidence to our hypothesized hydrologic formation mechanism, the presence or absence of such features cannot confirm nor nullify this mechanism. Sample MA113 shows clear textural evidence of formation in a quiet water body that is periodically disrupted by higher energy flushing events. Opal layers fill in low points and voids in the underlying calcite and have very flat tops (Supplementary Fig. 1a, c, d), which is strong indication that they formed by settling out of a water column. Opal layers are also entirely devoid of visible detritus, indicative of formation in a low energy system incapable of clastic delivery (Supplementary Fig. 1e, g). Conversely, many calcite layers have sand to silt-sized detritus formed during episodes of enhanced flushing energy (Supplementary Fig. 1a, c, d). In most cases detritus is concentrated at the bottom of the calcite layer pointing to flushing events ending the quiescent periods of opal growth and instigating calcite precipitation. These detritus-rich areas contain fine, <10μm-scale crystals, and give way to larger, detritus-free calcite growth of 100-1000μm-scale crystals above (Supplementary Fig. 1e, f).

Sample PRR50489 shows similar opal textures indicative of gravitational settling in a water column. However, detritus poor calcite suggests that the subglacial environment where this sample formed was much more sediment limited. However, at the base of many calcite layers, smaller crystals surround dark microdetritus or organic material that evinces hydrologic flushing and/or delivery of oxygen rich water (Supplementary Fig. 2b, c). At the tips of these layers, fibrous, clean calcite crystals grow and are eventually filled in with overlying opal.

Based on petrographic evidence of PRR50489 and MA113, we find no clear indication of diagenetic alteration in either of these subglacial precipitates. Aside from glaciotectonic disruption, opal-layers are relatively undisturbed and show no clear evidence for dissolution, which is borne out by XRD data showing that these layers remain a primary opal-A (Supplementary Fig. 3). Calcite layers are made of up delicate, fibrous crystals that also show no signs of dissolution or reprecipitation (Supplementary Fig. 1 and 2). We, therefore, regard diagenetic influence on our geochemical data to be insignificant.

**Supplementary Figures**

**
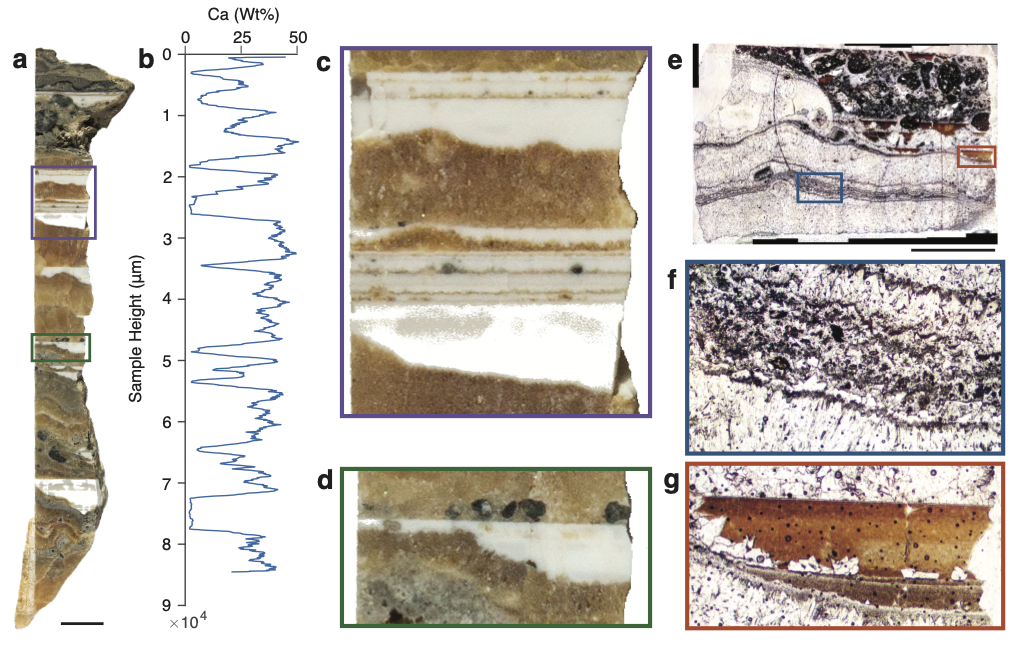
Supplementary Fig. 1 | Petrographic analysis of sample MA113. a.** Plain light image of sample MA113. **b.** Slab and SEM-EDS image of second piece of sample PRR50489. Purple and green boxes outline areas highlighted in c and d respectively. **b.** Plot of Ca concentration (wt. %) in MA113 versus sample height (μm). Spectra taken from top to bottom. **c.** Zoomed in image of area in MA113. Shows clean, white opal layers that fill voids space made by underlying calcite and have flat tops, indicative of formation from gravitational settling in a water column. **d.** Zoomed in image of lower area in MA113. Detrital grains lay at the base of calcite layers above opal layers. **e.** Thin section image of upper part of MA113 in plain polarized light. Blue and orange rectangles outline areas highlighted in f and g respectively. **f.** Zoomed in image of lower portion of a calcite layer. In this area of the sample dark, detrital grains can be seen in surrounded by calcite with smaller crystals. Above this part of the layer calcite becomes detritus free and exhibits larger crystals. **g.** Zoomed in image of opal layer in MA113. Opal is devoid of detritus and drapes calcite below it. Black scale bars represent 1 cm.

**
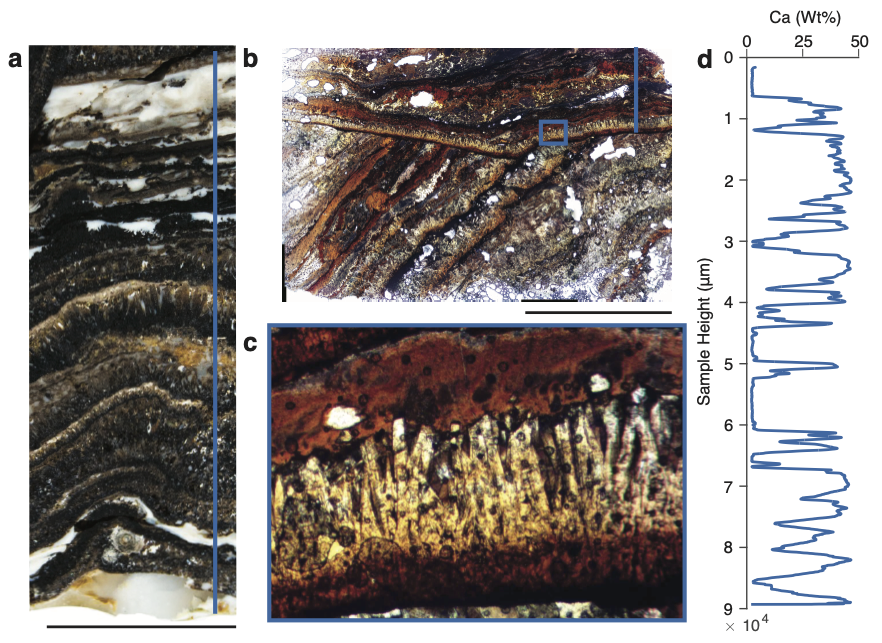
**

**Supplementary Fig. 2 | Petrographic analysis of sample PRR50489. a.** Plain light image of sample PRR50489. **b.** Thin section image of PRR50489 in plain polarized light. Blue box represents area shown in c. **c.** Zoomed in image of single calcite and opal layer. Calcite layer is bladed, with no clear indication of alteration or dissolution. Bottom of calcite layer characterized by smaller crystal size and darker color, which we interpret to be micro detritus or organic matter. Opal fills gaps in calcite crystals and drapes the calcite layer. **d.** Plot of Ca concentration (wt. %) in PRR50489 versus sample height (μm). Area where spectra were taken is represented by blue line in a and b. Spectra is taken from bottom of sample in a, and continues from bottom to top of d. Black scale bars represents 1 cm.

**Supplementary Fig. 3 | XRD from PRR50489 opal. a.** Powder XRD patterns from PRR50489 opal. This sample was not leached to remove calcite prior to sampling. So XRD patterns include both calcite and opal. **b.** Calcite XRD pattern output from Profex XRD reduction software. Peaks in calcite spectra clearly represent steepest peaks in PRR50489 spectra. **c**. Opal-A XRD pattern output from RRUF database. The largest peak at 23˚ clearly aligns with a similar peak in PRR50489 spectra.


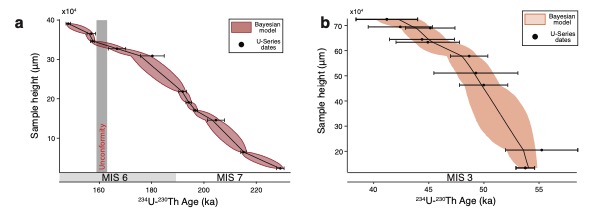


**Supplementary Fig. 4 | Stratigraphic Age models. a.** Height versus age curve for PRR50489. Position of angular unconformity marker in grey. **b.** Height versus age curve for MA113. Black markers are ^234^U-^230^Th with 2σ error bars. Red and orange envelopes are Bayesian age-depth model using stratigraphic position as a prior to refine dating uncertainties^66^.

**Supplementary Fig. 5 | Rare Earth Element Ternary Plot.** Ternary plot with Rare Earth Elements gadolinium, neodymium, and ytterbium. Filled areas plot compositional range for different surface and groundwaters after ref.^67^. Triangles represent Lake Vanda water compositions colored based on depth from 57m to 67m depth^68^. PRR50489 calcite and opal compositions are plotted as black and blue spheres respectively. PRR50489 data were collected using LA ICP-MS.

**Supplementary Fig. 6 |** **Model simulations predicting opal formation from CaCl_2_** **brine.** PHREEQC simulations of cryoconcentration of Don Juan Pond (DJP) CaCl_2_ brine^69,70^ over a range of temperatures and relative concentrations. X-axes show the relative fraction of water that enters the system (as meltwater) or leaves the system (as ice) upon equilibration of the brine with the overlying ice. Fraction water Δ values above 1 indicate meltwater addition; fraction water Δ values below indicate water loss via freezing. Plots show saturation indices (SI) — the log ratio of the ion activity product and equilibrium constant — of ice and opal. SI values greater than 0 are supersaturated with respect to that phase; values less than 0 are undersaturated. The grey, dashed lines delineate SI precipitation threshold for opal and ice. Saturation indices of calcite are not shown because the lack of carbon in DJP brine makes the solution unsaturated with respect to calcite **a.** Equilibration of concentrated DJP brine with ice and opal over a range of temperatures between -5 and 5˚C. High ionic strengths in the concentrated brine causes significant melting of overlying ice, diluting the solution with respect to opal, and inhibiting opal precipitation. **b.** Equilibration of 10x diluted DJP brine with ice and opal over a range of temperatures between -5 and 5˚C. In this case the brine starts to freeze at -2˚C, but the solution does not reach opal precipitation because the degree of freezing (i.e., cryoconcentration) of Si is suppressed by the high ionic strength of the brine. **c.** Equilibration of 50x diluted DJP brine with ice and opal over a range of temperatures between -5 and 5˚C. This solution also reaches freezing at -2˚C, but the relatively lower ionic strength allows more significant portions of cryoconcentration of Si, causing opal to precipitate near -3.5˚C **d.** Equilibration of 50x diluted DJP brine with ice and opal over a smaller range of temperatures, between -4 and -3˚C. This plot shows that opal precipitation occurs when ~75% of the water is lost via freezing, which occurs at ~ -3.5˚C.

**Supplementary Fig. 7 |** **Model simulations predicting calcite formation via mixing of meltwater and CaCl_2_ brine.** PHREEQC simulations of a range of mixing ratios between CaCl_2_ brine and Casey Station jökulhlaup water. Brine starting temperature is set at -3.5˚C matching ideal conditions for opal precipitation shown in Extended Data figure 5; meltwater starting temperature is at -1.5˚C based on the amount of heat added to the system by shear heating in our reduced complexity ice sheet model. Calcite precipitation threshold is defined by the observation that calcite precipitation can be inhibited until strong supersaturation^72,73^. Plot shows that meltwater addition halts opal precipitation, while generating calcite precipitation in a mixing ratio between 30:70 and 80:20 meltwater to brine.

 **Supplementary Fig. 8 |** **﻿Long-term results of measurements of NBS 4321 (5.2919 × 10^−5^± 0.013 × 10^−5^ (0.25%)) at UCSC using an IsotopX X62, TIMS. All uncertainties are absolute 2σ.**


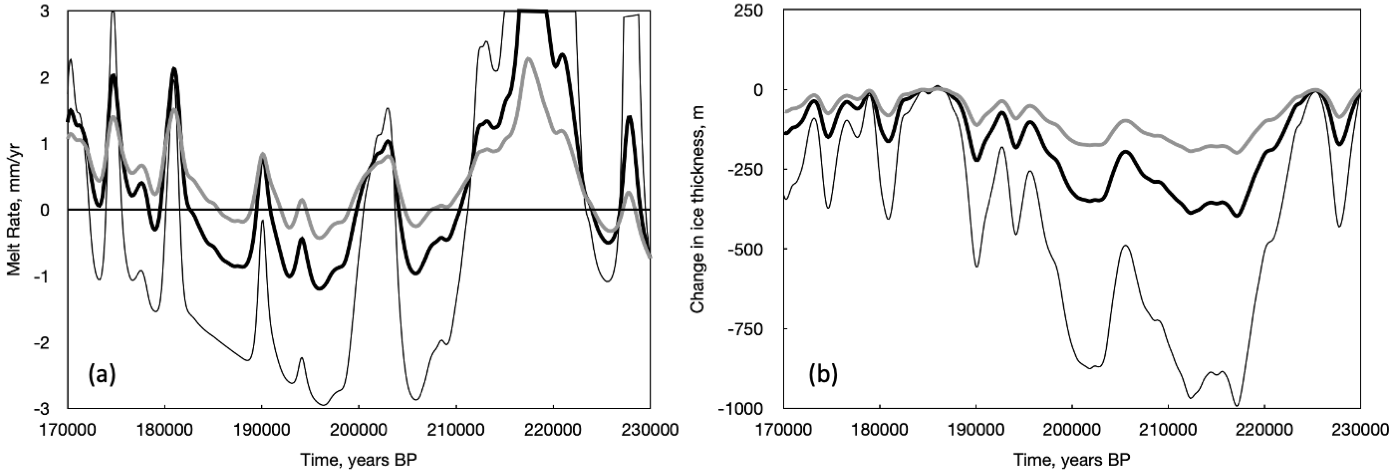


**Supplementary Figure 9** **| RCMIST Outputs for Sample PRR50489. a.** Sensitivity of the basal thermal energy balance, expressed in terms of equivalent basal melt (+) or freeze (-) rate given in mm/year. The thick black line shows the preferred scenario for $C_{i}$ = 10 m per ‰ of δD from the EDC ice core record. The thin black line gives the $C_{i}$ = 25 scenario and the thick grey line is for the $C_{i}$ = 5 case. **b.** Equivalent changes in ice thickness, $H_{RE}\left( t \right)$, which represent the impact of climate forcing on our model through equation 4. The same types of lines as in **a.** are used here to represent the three cases $C_{i}=$ 5, 10 and 25 m per ‰.


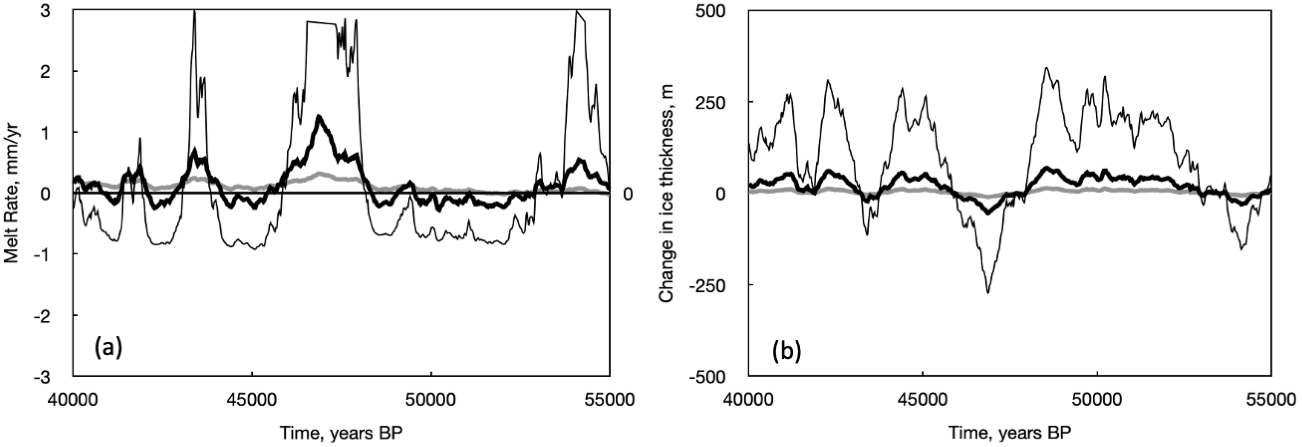


**Supplementary Figure 10** **| Reduced Complexity Ice Sheet Model Outputs for Sample MA113.** Plots equivalent to those in Supplementary Figure 1 but for simulations pertaining to the sample MA113 and with $C_{i}=$ 10, 50, and 250 of m per ‰ of δ^18^O, corresponding to the thick grey lines, **a.** Sensitivity of the basal thermal energy balance, expressed in terms of equivalent basal melt (+) or freeze (-) rate given in mm/year. The thick black lines show the preferred scenario for $C_{i}$ = 50 m per ‰ of d^18^O from the WAIS Divide ice core record. The thin black lines give the $C_{i}$ = 250 scenario and the thick grey lines are for the $C_{i}$ = 10 case. **b.** Equivalent changes in ice thickness, $H_{RE}\left( t \right)$, which represent the impact of climate forcing on our model through equation 4. The same types of lines as in **a.** are used here to represent the three cases $C_{i}=$ 5, 10 and 25 m per ‰.

**Supplementary References**

1. Condon, D. J., McLean, N., Noble, S. R. & Bowring, S. A. Isotopic composition (238U/235U) of some commonly used uranium reference materials. *Geochim. Cosmochim. Acta* **74**, 7127–7143 (2010).

2. Hamelin, B., Bard, E., Zindler, A. & Fairbanks, R. G. 234U238U mass spectrometry of corals: How accurate is the UTh age of the last interglacial period? *Earth Planet. Sci. Lett.* **106**, 169–180 (1991).

3. Frisia, S. *et al.* The influence of Antarctic subglacial volcanism Maximum. *Nat. Commun.* **8**, 1–9 (2017).

4. Cheng, H. *et al.* The half-lives of uranium-234 and thorium-230. *Chem. Geol.* **169**, 17–33 (2000).

5. Jamieson, S. S. R., Sugden, D. E. & Hulton, N. R. J. The evolution of the subglacial landscape of Antarctica. *Earth Planet. Sci. Lett.* **293**, 1–27 (2010).

6. Skidmore, M., Tranter, M., Tulaczyk, S. & Lanoil, B. Hydrochemistry of ice steams beds- evaporitic or microbial effects? *Hydrol. Process.* **24**, 517–523 (2010).

7. Kassab, C. M. *et al.* Formation and evolution of an extensive blue ice moraine in central Transantarctic Mountains, Antarctica. *J. Glaciol.* **66**, 49–60 (2019).

8. Bader, N. A., Licht, K. J., Kaplan, M. R., Kassab, C. & Winckler, G. East Antarctic ice sheet stability recorded in a high-elevation ice-cored moraine. *Quat. Sci. Rev.* **159**, 88–102 (2017).

9. Graly, J. A., Licht, K. J., Kassab, C. M., Bird, B. W. & Kaplan, M. R. Warm-based basal sediment entrainment and far-field Pleistocene origin evidenced in central Transantarctic blue ice through stable isotopes and internal structures. *J. Glaciol.* **64**, 185–196 (2018).

10. Faure, G. Physical Description of the Elephant and Reckline Moraines. in *Workshop on Antarctic Meteorite Standing Surfaces* 24–25 (1990).

11. Cassidy, W., Harvey, R., Schutt, J., Delise, G. & Yanai, K. The meteorite collection of Antarctica. *Meteorites* **27**, 490–525 (1992).

12. Bintanja, R. On the Glaciological, Meteorological, and Significance. *Rev. Geophys.* **37**, 337–359 (1999).

13. Rhee, H. H. *et al.* Quaternary ice thinning of David Glacier in the Terra Nova Bay region, Antarctica. *Quat. Geochronol.* **67**, 101233 (2022).

14. Faure, G. & Harwood, D. M. Marine microfossils in till clasts of the Elephant Moraine on the east antarctic ice sheet. in *Antarctic Journal of the United States* 23–25 (1990).

15. Fretwell, P. *et al.* Bedmap2: Improved ice bed, surface and thickness datasets for Antarctica. *Cryosphere* **7**, 375–393 (2013).

16. Matsuoka, K. *et al.* Quantarctica, an integrated mapping environment for Antarctica, the Southern Ocean, and sub-Antarctic islands. *Environ. Model. Softw.* **140**, (2021).

17. Nishiizumi, K. Subaerial exposure ages of bedrock near meteorite stranding surfaces. in *Antarctic Meteorite Stranding Surfaces* 60–64 (1990).

18. Jull, A. J. T. Terrestrial ages of meteorites. in *Accretion of extraterrestrial matter throughout Earth’s history* 241–266 (2001). doi:10.1038/293433a0

19. Coren, F., Delisle, G. & Sterzai, P. Ice dynamics of the Allan Hills meteorite concentration sites revealed by satellite aperture radar interferometry. *Meteorit. Planet. Sci.* **38**, 1319–1330 (2003).

20. Rignot, E., Mouginot, J. & Scheuchl, B. Ice flow of the antarctic ice sheet. *Science (80-. ).* **333**, 1427–1430 (2011).

21. Mouginot, J., Scheuch, B. & Rignot, E. Mapping of ice motion in antarctica using synthetic-aperture radar data. *Remote Sens.* **4**, 2753–2767 (2012).

22. Fudge, T. J. *et al.* Variable relationship between accumulation and temperature in West Antarctica for the past 31,000 years. *Geophys. Res. Lett.* **43**, 3795–3803 (2016).

23. Yan, Y. *et al.* Two-million-year-old snapshots of atmospheric gases from Antarctic ice. *Nature* **574**, (2019).

24. Souchez, R., Lemmens, M. & Chappellaz, J. Flow‐induced mixing in the GRIP basal ice deduced from the CO2 and CH4 records. *Geophys. Res. Lett.* **22**, 41–44 (1995).

25. Montross, S. *et al.* Debris-Rich Basal Ice as a Microbial Habitat, Taylor Glacier, Antarctica. *Geomicrobiol. J.* **31**, 76–81 (2014).

26. Steig, E. J. *et al.* Continuous-Flow Analysis of δ 17O, δ 18O, and δD of H2O on an Ice Core from the South Pole. *Front. Earth Sci.* **9**, 1–14 (2021).

27. Jouzel, Jean and Souchez, R. A. Melting-Refreezing At the Glacier Sole and the Isotopic Composition of Ice. *J. Glaciol.* **28**, 35–42 (1982).

28. Faure, G., Hoefs, J., Jones, L. M., Curtis, J. B. & Pride, D. E. Extreme 18O depletion in calcite and chert clasts from the Elephant Moraine on the East Antarctic ice sheet. *Nature* **332**, 352–354 (1988).

29. Graly, J. A., Drever, J. I. & Humphrey, N. F. Calculating the balance between atmospheric CO2 drawdown and organic carbon oxidation in subglacial hydrochemical systems. *Global Biogeochem. Cycles* **31**, 709–727 (2017).

30. Marsh, N. B. *et al.* Sources of solutes and carbon cycling in perennially ice-covered Lake Untersee, Antarctica. *Sci. Rep.* **10**, 1–12 (2020).

31. Diaz, M. A. *et al.* Stable Isotopes of Nitrate, Sulfate, and Carbonate in Soils From the Transantarctic Mountains, Antarctica: A Record of Atmospheric Deposition and Chemical Weathering. *Front. Earth Sci.* **8**, 1–19 (2020).

32. Lorius, C., Merlivat, L., Jouzel, J. & Pourchet, M. A 30,000-yr isotope climatic record from Antarctic ice. *Nature* **280**, 644–648 (1979).

33. Aciego, S., Bourdon, B., Schwander, J., Baur, H. & Forieri, A. Toward a radiometric ice clock: Uranium ages of the Dome C ice core. *Quat. Sci. Rev.* **30**, 2389–2397 (2011).

34. Kigoshi, K. Alpha-Recoil Thorium-234 : Dissolution into Water and the Uranium-234 / Uranium-238 Disequilibrium in Nature. *Science (80-. ).* **173**, 47–48 (1971).

35. Lyons, W. B. *et al.* The Geochemistry of Englacial Brine From Taylor Glacier, Antarctica. *J. Geophys. Res. Biogeosciences* **124**, 633–648 (2019).

36. Henderson, G. M., Hall, B. L., Smith, A. & Robinson, L. F. Control on (234U/238U) in lake water: A study in the Dry Valleys of Antarctica. *Chem. Geol.* **226**, 298–308 (2006).

37. Fitzpatrick, J. J., Muhs, D. R. & Jull, A. J. T. Saline Minerals in the Lewis Cliff Ice Tongue, Buckley Island Quadrangle, Antarctica. *Contrib. to Antarct. Res. I* **50**, 57–69 (1990).

38. Blackburn, T. *et al.* Ice retreat in Wilkes Basin of East Antarctica during a warm interglacial. *Nature* **583**, 554–559 (2020).

39. Goodwin, I. D. *et al.* Modern to Glacial age subglacial meltwater drainage at Law Dome, coastal East Antarctica from topography, sediments and jökulhlaup observations. *Geol. Soc. Spec. Publ.* **461**, 215–230 (2018).

40. Refsnider, K. A. *et al.* Subglacially precipitated carbonates record geochemical interactions and pollen preservation at the base of the Laurentide Ice Sheet on central Baffin Island, eastern Canadian Arctic. *Quat. Res.* **81**, 94–105 (2014).

41. Blackburn, T. *et al.* Composition and formation age of amorphous silica coating glacially polished surfaces. *Geology* **47**, 347–350 (2019).

42. MacAyeal, D. R. Binge/purge oscillations of the Laurentide Ice Sheet as a cause of the North Atlantic’s Heinrich events. *Paleoceanography* **8**, 775–784 (1993).

43. Morse, D. L., Waddington, E. D. & Rasmussen, L. A. Ice deformation in the vicinity of the ice-core site at Taylor Dome, Antarctica, and a derived accumulation rate history. *J. Glaciol.* **53**, 449–460 (2007).

44. Favier, V. *et al.* An updated and quality controlled surface mass balance dataset for Antarctica. *Cryosphere* **7**, 583–597 (2013).

45. Hulbe, C. & Fahnestock, M. Century-scale discharge stagnation and reactivation of the Ross ice streams, West Antarctica. *J. Geophys. Res. Earth Surf.* **112**, 1–11 (2007).

46. Tulaczyk, S., Kamb, W. B. & Engelhardt, H. F. Basal mechanics of Ice Stream B, West Antarctica 2. Undrained plastic bed model. *J. Geophys. Res. Solid Earth* **105**, 483–494 (2000).

47. Goodge, J. W. Crustal heat production and estimate of terrestrial heat flow in central East Antarctica, with implications for thermal input to the East Antarctic ice sheet. *Cryosphere* **12**, 491–504 (2018).

48. Blasco, J., Tabone, I., Alvarez-Solas, J., Robinson, A. & Montoya, M. The Antarctic Ice Sheet response to glacial millennial-scale variability. *Clim. Past* **15**, 121–133 (2019).

49. Barbante, C. *et al.* One-to-one coupling of glacial climate variability in Greenland and Antarctica. *Nature* **444**, 195–198 (2006).

50. Buizert, C. *et al.* The WAIS Divide deep ice core WD2014 chronology &ndash; Part 1: Methane synchronization (68-31 ka BP) and the gas age-ice age difference. *Clim. Past* **11**, 153–173 (2015).

51. Begeman, C. B., Tulaczyk, S. M. & Fisher, A. T. Spatially Variable Geothermal Heat Flux in West Antarctica: Evidence and Implications. *Geophys. Res. Lett.* **44**, 9823–9832 (2017).

52. Cauquoin, A. *et al.* Comparing past accumulation rate reconstructions in East Antarctic ice cores using 10Be, water isotopes and CMIP5-PMIP3 models. *Clim. Past* **11**, 355–367 (2015).

53. Bintanja, R. On the Glaciological, Meteorological, and Climatological Significance of Antarctic Blue Ice Areas. *Rev. Geophys.* **37**, 337–359 (1999).

54. Cuffey, K. M. & Paterson, W. S. . B. *Physics of Glaciers, Fourth Edition*. *The Physics of Glaciers* (2010).

55. Ritz, C. Time dependent boundary conditions for calculation of temperature fields in ice sheets. *Int. Assoc. Hydrol. Sci.* **170**, 207–216 (1987).

56. Goehring, B. M., Balco, G., Todd, C., Moening-Swanson, I. & Nichols, K. Late-glacial grounding line retreat in the northern Ross Sea, Antarctica. *Geology* **47**, 291–294 (2019).

57. Spector, P. *et al.* Rapid early-Holocene deglaciation in the Ross Sea, Antarctica. *Geophys. Res. Lett.* **44**, 7817–7825 (2017).

58. Neuhaus, S. *et al.* Did Holocene climate changes drive West Antarctic grounding line retreat and re-advance? *Cryosph. Discuss.* 1–30 (2021). doi:10.5194/tc-2020-308

59. Members, W. D. P. Precise interpolar phasing of abrupt climate change during the last ice age. *Nature* **520**, 661–665 (2015).

60. Jouzel, J. *et al.* Orbital and millennial antarctic climate variability over the past 800,000 years. *Science (80-. ).* **317**, 793–796 (2007).

61. Veres, D. *et al.* The Antarctic ice core chronology (AICC2012): An optimized multi-parameter and multi-site dating approach for the last 120 thousand years. *Clim. Past* **9**, 1733–1748 (2013).

62. Nye, J. F. The response of glaciers and ice-sheets to seasonal and climatic changes. *Proc. R. Soc. London. Ser. A. Math. Phys. Sci.* **256**, 559–584 (1960).

63. Cuffey, K. M. & Paterson, W. S. B. *The physics of glaciers*. (2010).

64. Siddall, M., Rohling, E. J., Thompson, W. G. & Waellbroeck, C. Marine isotope stage 3 sea level fluctuations: Data synthesis and new outlook. *Rev. Geophys.* **46**, 1–29 (2008).

65. Anderson, J. B. *et al.* Ross Sea paleo-ice sheet drainage and deglacial history during and since the LGM. *Quat. Sci. Rev.* **100**, 31–54 (2014).

66. Keller, B. A Bayesian framework for integrated eruption age and age-depth modelling. (2020). doi:doi:10.17605/OSF.IO/TQX3F

67. Martin, J. E., Patrick, D., Kihm, A. J., Foit, F. F. & Grandstaff, D. E. Lithostratigraphy, tephrochronology, and rare earth element geochemistry of fossils at the classical pleistocene Fossil Lake area, south central Oregon. *J. Geol.* **113**, 139–155 (2005).

68. De Carlo, E. H. & Green, W. J. Rare earth elements in the water column of Lake Vanda, McMurdo Dry Valleys, Antarctica. *Geochim. Cosmochim. Acta* **66**, 1323–1333 (2002).

69. Green, W. J. & Canfield, D. E. Geochemistry of the Onyx River (Wright Valley, Antarctica) and its role in the chemical evolution of Lake Vanda. *Geochim. Cosmochim. Acta* **48**, 2457–2467 (1984).

70. Harris, H. J. H. & Cartwright, K. Hydrology of the Don Juan Basin, Wright Valley, Antarctica. *Antarct. Res. Ser.* **33**, 162–184 (1981).

71. Tréguer, P. *et al.* The silica balance in the world ocean: A reestimate. *Science (80-. ).* **268**, 375–379 (1995).

72. Dreybrodt, W., Buhmann, D., Michaelis, J. & Usdowski, E. Geochemically controlled calcite precipitation by CO2 outgassing: Field measurements of precipitation rates in comparison to theoretical predictions. *Chem. Geol.* **97**, 285–294 (1992).

73. Dandurand, J. L. *et al.* Kinetically controlled variations of major components and carbon and oxygen isotopes in a calcite-precipitating spring. *Chem. Geol.* **36**, 299–315 (1982).
