# Supplementary material for: Subglacial precipitates record Antarctic ice sheet response to late Pleistocene millennial climate cycles
Source: Nat Commun. 2022 Sep 15;13:5428. doi: 10.1038/s41467-022-33009-1 (PMC9477832; doi:10.1038/s41467-022-33009-1)
Supplement: Supplementary file 3 — Reporting Summary [file 41467_2022_33009_MOESM3_ESM.pdf]

nature portfolio

Corresponding author(s): Gavin Piccione

Last updated by author(s): Aug 6, 2022

Reporting Summary

Nature Portfolio wishes to improve the reproducibility of the work that we publish. This form provides structure for consistency and transparency in reporting. For further information on Nature Portfolio policies, see our Editorial Policies and the Editorial Policy Checklist.

Statistics

For all statistical analyses, confirm that the following items are present in the figure legend, table legend, main text, or Methods section.

n/a	Confirmed	
☐	☒	The exact sample size (*n*) for each experimental group/condition, given as a discrete number and unit of measurement
☐	☒	A statement on whether measurements were taken from distinct samples or whether the same sample was measured repeatedly
☒	☐	The statistical test(s) used AND whether they are one- or two-sided<br>*Only common tests should be described solely by name; describe more complex techniques in the Methods section.*
☒	☐	A description of all covariates tested
☐	☒	A description of any assumptions or corrections, such as tests of normality and adjustment for multiple comparisons
☐	☒	A full description of the statistical parameters including central tendency (e.g. means) or other basic estimates (e.g. regression coefficient) AND variation (e.g. standard deviation) or associated estimates of uncertainty (e.g. confidence intervals)
☒	☐	For null hypothesis testing, the test statistic (e.g. *F*, *t*, *r*) with confidence intervals, effect sizes, degrees of freedom and *P* value noted<br>*Give P values as exact values whenever suitable.*
☐	☒	For Bayesian analysis, information on the choice of priors and Markov chain Monte Carlo settings
☒	☐	For hierarchical and complex designs, identification of the appropriate level for tests and full reporting of outcomes
☒	☐	Estimates of effect sizes (e.g. Cohen's *d*, Pearson's *r*), indicating how they were calculated

Our web collection on statistics for biologists contains articles on many of the points above.

Software and code

Policy information about availability of computer code

Data collection	No software were used to collect data in this study.
Data analysis	LA ICP-MS data were analyzed using Iolite software version 4; U-series data were analyzed using an in-house Matlab code to apply initial Th corrections and output ages; Age-depth models were constructed using CHRON: a published Bayesian framework that applies the principal of stratigraphic superposition as a prior; Precipitate mineralogy timeseries were constructed using an in-house Monte Carlo approach to find the optimal fit with ice core climate proxies.

For manuscripts utilizing custom algorithms or software that are central to the research but not yet described in published literature, software must be made available to editors and reviewers. We strongly encourage code deposition in a community repository (e.g. GitHub). See the Nature Portfolio guidelines for submitting code & software for further information.

Data

Policy information about availability of data

All manuscripts must include a data availability statement. This statement should provide the following information, where applicable:

- Accession codes, unique identifiers, or web links for publicly available datasets
- A description of any restrictions on data availability
- For clinical datasets or third party data, please ensure that the statement adheres to our policy

The U-series and isotopic data generated in this study have been deposited in the US Antarctic program database under accession code ZZ [add hyperlink here. All

data generated in this study are also provided in the Supplementary Information Data Tables 1–4. Sample PRR50489 was provided by the US Polar Rock Repository at the. Byrd Polar and Climate Research Center at Ohio State University, and can be accessed at https://prr.osu.edu/collection/object/100478

Human research participants

Policy information about studies involving human research participants and Sex and Gender in Research.

Reporting on sex and gender	N/A
Population characteristics	N/A
Recruitment	N/A
Ethics oversight	N/A

Note that full information on the approval of the study protocol must also be provided in the manuscript.

Field-specific reporting

Please select the one below that is the best fit for your research. If you are not sure, read the appropriate sections before making your selection.

☐ Life sciences ☐ Behavioural & social sciences ☒ Ecological, evolutionary & environmental sciences

For a reference copy of the document with all sections, see nature.com/documents/nr-reporting-summary-flat.pdf

Ecological, evolutionary & environmental sciences study design

All studies must disclose on these points even when the disclosure is negative.

Study description	We measured the age, isotopic, and elemental composition of 32 layers within two subglacial precipitate samples from Antarctica. We paired these data with models of the precipitation mechanism, climate proxies, and the thermodynamics of the subglacial environment to describe how these data linked climate change to ice sheet velocity changes.
Research sample	Sample PRR50489 is a subglacial precipitate from Elephant moraine, Antarctica. It is an ~3 cm rock made of layers of calcite and opal. MA113 is a subglacial precipitate sample from Law glacier, Antarctica. It was formed over time between 228 and 148 kyr ago. It was loaned by the US Polar Rock Repository. It is an ~7cm rock made of layers of calcite and opal. It was formed over time between 54 and 25 kyr. It was provided by Dr. Kathy Licht, who collected it. These samples were chosen for this study because they provide long-term records of opal-calcite transitions beneath the Antarctic Ice Sheet.
Sampling strategy	For U-series data we sampled each layer at least once and were able to measure ages on samples with sufficient concentration of uranium, and sufficiently low concentrations of thorium. This sample size, paired with the achieved levels of uncertainty, provided us with an age-depth model with precision high enough to assess millennial-scale changes in the subglacial hydrologic system. Isotopic analyses were done on each sample layer, and provide signal:noise ratio >1000.
Data collection	U-series data and Sr isotopic data were collected on the Thermal Ionization Mass Spectrometer at the University of California Santa Cruz by GP and TB. LA ICP-MS data were measured on the Agilent Quadrapole Mass Spectrometer, coupled with a New Wave 213nm laser at Stony Brook University. LA ICP-MS data were collected by TR. Stable Isotope Analyses were measured on a Kiel at UCSC by Colin Carney (laboratory technician at the UCSC stable isotope laboratory.) Opal oxygen isotope data were measured on a Thermo Scientific MAT 253+ dual-inlet isotope ratio mass spectrometer by DI.
Timing and spatial scale	Data were collected continuously between February of 2020 and December of 2021.
Data exclusions	U-series data with insufficient signal to noise ratios were excluded, as they cannot provide meaningful information about the sample age. Sr data that did not have a sufficient level of measurement precision were also excluded and re-run using archive sample halves.
Reproducibility	Stable isotopes reproducibility was referenced δ13CCO3 and δ18OCO3 to Vienna PeeDee Belemnite (VPDB) is calculated by two-point correction to externally calibrated Carrara Marble 'CM12' and carbonatite NBS-1874. Externally calibrated coral 'Atlantis II'75 was measured for independent quality control. Typical reproducibility of replicates was significantly better than 0.05 ‰ for δ13CCO3 and 0.1 ‰ for δ18OCO3. Opal δ18O values were referenced to Three secondary standards: BX-88 (Stanford Laboratory internal standard), UCD-DFS and PS1772-8 were also analyzed over the course of the analyses. Accuracy of the 87Sr/86Sr measurements is evaluated using Sr standard SRM987 compared to a long-term laboratory average value of 0.71024, with a typical reproducibility of ±0.00004. Accuracy of the U-series measurements was evaluated using Uranium standard NBS4321
Randomization	All data for the two samples in this study were grouped based on the sample, and whether the layers measured were opal or calcite.
Blinding	Blinding is not necessary for these analyses.

Did the study involve field work? ☐ Yes ☒ No

Reporting for specific materials, systems and methods

We require information from authors about some types of materials, experimental systems and methods used in many studies. Here, indicate whether each material, system or method listed is relevant to your study. If you are not sure if a list item applies to your research, read the appropriate section before selecting a response.

Materials & experimental systems

n/a	Involved in the study
☒	☐ Antibodies
☒	☐ Eukaryotic cell lines
☒	☐ Palaeontology and archaeology
☒	☐ Animals and other organisms
☒	☐ Clinical data
☒	☐ Dual use research of concern

Methods

n/a	Involved in the study
☒	☐ ChIP-seq
☒	☐ Flow cytometry
☒	☐ MRI-based neuroimaging
